# Childhood Conscientiousness and Leukocyte Telomere Length 40 Years Later in Adult Women—Preliminary Findings of a Prospective Association

**DOI:** 10.1371/journal.pone.0134077

**Published:** 2015-07-28

**Authors:** Grant W. Edmonds, Hélène C. F. Côté, Sarah E. Hampson

**Affiliations:** 1 Oregon Research Institute, Eugene, Oregon, United States of America; 2 Department of Pathology & Laboratory Medicine, University of British Columbia, Vancouver, BC, Canada; Université de Montréal, CANADA

## Abstract

Leukocyte telomere length (LTL) shortens with age, and is a prospective marker of mortality related to cardiovascular disease. Many health behaviors and social environmental factors have been found to be associated with LTL. Several of these are also associated with conscientiousness, a dispositional personality trait. Conscientiousness is a propensity to be planful, adhere to social norms, and inhibit pre-potent responses. Like LTL, conscientiousness is prospectively related to mortality, possibly through cumulative effects on health over the life course via multiple pathways. As a result, we hypothesized that childhood levels of conscientiousness would predict LTL prospectively in adulthood. We selected a sample of 60 women in the Hawaii Personality and Health Cohort; 30 described by their teachers as high on conscientiousness in childhood and 30 described as low on the trait. Dried blood spot samples collected in adulthood 40 years later were used as sources of DNA for the LTL assay. Conscientiousness was associated with longer LTL (*p* = .02). Controlling for age did not account for this association. Controlling for education and physiological dysregulation partially attenuated the association, and the effect remained significant when accounting for differences in LTL across cultural groups. These results represent the first evidence that childhood personality prospectively predicts LTL 40 years later in adulthood. Our findings would be consistent with a mediation hypothesis whereby conscientiousness predicts life paths and trajectories of health that are reflected in rates of LTL erosion across the lifespan.

## Introduction

A considerable amount of evidence has accumulated demonstrating that personality traits show important associations with health and longevity [1]. Conscientiousness, a dispositional tendency to be industrious, norm adhering, planful, and to thoughtfully inhibit impulses [2], has emerged as the Big Five trait most consistently associated with these outcomes [3,4]. Mechanisms linking conscientiousness to health are diverse and include health behaviors such as smoking, physical activity, and diet [5]. Conscientiousness also operates on health outcomes via social environmental factors, such as educational attainment [6], and socioeconomic status [7]. As a result, conscientiousness has the potential to offer a large cumulative health benefit across multiple pathways, which may account for the now well-established association between conscientiousness and mortality [3]. Conscientiousness is related to patterns of states and behaviors across a wide variety of health related domains, and as a result plays an important role in models of successful aging [8].

Many of the same mechanisms linking conscientiousness to health and mortality, including physical activity, smoking, and educational attainment, have also been found to predict leukocyte telomere length (LTL) [9–12]. Telomeres are nucleoprotein structures which cap the ends of chromosomes, maintaining their structural integrity and preventing end to end fusion [13]. In the absence of telomerase activity, telomeres shorten with each cell division [14]. Critically short telomere length has been proposed as a mechanism leading to cell senescence or cell death [15]. As a result, LTL has been proposed as a marker of biological cell aging [16, 17]. Consistent with this, shorter LTL is prospectively associated with mortality from cardiovascular disease in elderly individuals [18, 19]. Evidence for LTL as causal mechanism in the aging process however is limited, and at the present time it is perhaps best viewed as a surrogate endpoint for cumulative cellular damage [20].

Because LTL is a cumulative outcome resulting from biological insults and stresses across many years, dispositional traits, which may exert persistent effects on factors related to health, are expected to also be associated with LTL [21]. While this view is generally accepted, most of the work to date focusing on dispositional characteristics and variation in LTL has focused on health damaging effects associated with depression [22], anxiety [23], and neuroticism [21] in the context of stressful and traumatic life events [24]. In contrast, few studies have considered that dispositional traits positively associated with health might be associated LTL. Despite a large body of work suggesting overlapping correlates across both conscientiousness and LTL, only one recent study in Japanese medical students (mean age 23) has reported a cross sectional association between longer LTL and conscientiousness [25]. To our knowledge ours is the first study investigating a prospective association between conscientiousness in childhood and LTL in later life. Predicated on the notion that conscientiousness serves as a stable dispositional trait which increases the likelihood that individuals will participate in a variety of health promoting behaviors and select into social environments that are conducive to health, we propose that conscientiousness will have a protective effect on LTL. Accordingly, we hypothesized that conscientiousness will predict LTL prospectively, such that individuals higher on conscientiousness early in life will show longer LTL in adulthood. We present pilot results using data from the ongoing Hawaii Personality and Health Study. A small number of stored dried blood samples out of a pool of approximately 800 available for testing were selected to validate our methods of DNA extraction and assaying LTL; we additionally planned to use these pilot data for an initial test of the association between childhood conscientiousness and LTL. Previously we had demonstrated that childhood conscientiousness predicts a global measure of physical dysregulation comprised of 11 objective biomarkers [26]. As LTL and physiological dysregulation may be complimentary outcomes, we tested the degree to which childhood conscientiousness was associated with LTL independent of physiological dysregulation. Because LTL has been demonstrated to vary across cultural and ethnic groups [27, 28], we additionally report on differences in LTL across ethnic and cultural groups and include this important variable in our analyses.

## Methods

### Ethics Statement

Study protocols for locating participants in adulthood, the adult clinic visit and for follow-up questionnaires in adulthood were approved by the IRBs at both the Oregon Research Institute and the Kaiser Permanente Center for Health Research, Hawaii. Upon being contacted in adulthood, the participants included in these analyses gave consent for their childhood personality data to be used as part of an ongoing longitudinal study. Written consent was provided prior to the clinic visit in adulthood, and participants who agreed to provide blood samples gave additional written consent for these to be used for future genetic analyses. Prior to completing questionnaires in adulthood, all participants completed and returned written consent forms by mail.

### Participants

We used existing data and stored dried blood spots from the Hawaii Longitudinal Personality and Health Study. This project began with teacher assessments of childhood personality between 1959 and 1967 when the child sample was an average age of 10. Elementary school teachers’ personality assessments of all the children in their classrooms were obtained from elementary schools on the Hawaiian islands of Oahu and Kauai (*n* = 2,418). The schools were geographically dispersed across these islands, and represented a wide range of socio-economic status (SES).

Beginning in 1998, efforts were initiated to locate members of the original child cohort in adulthood [29]. Currently, 84% of the 2,320 available participants have been located. Of the 1,904 individuals who were living and available at the time of initial contact, 1,387 (73%) have agreed to participate in further research. Participants have completed several questionnaires administered between 1999 and the present. Data from two questionnaires were used in this report. Objective measures of physical health were collected as part of a clinic visit conducted at the Kaiser Permanente Center for Health Research, Hawaii in Honolulu, and also in clinics on the islands of Kauai and Hawaii. Health assessments were conducted by qualified staff using standard protocols, and included the collection of dried blood spots (see below for details). A total of 807 participants completed the clinic visit between 2002 and 2011, and 699 of these participants agreed to provide dried blood samples. The average year of assessment was 2006; approximately 40 years after the childhood assessments. The mean age of the clinic sample at the time of assessment was 51 years. The full adult sample reflects the ethnic diversity of Hawaii, and includes 44% Asian Americans, 22% Native Hawaiians or part Native Hawaiians, 15% of European ancestry, and 11% of Filipino ancestry, 4% indicating “other”, and 4% declining to respond.

**Fig 1:**
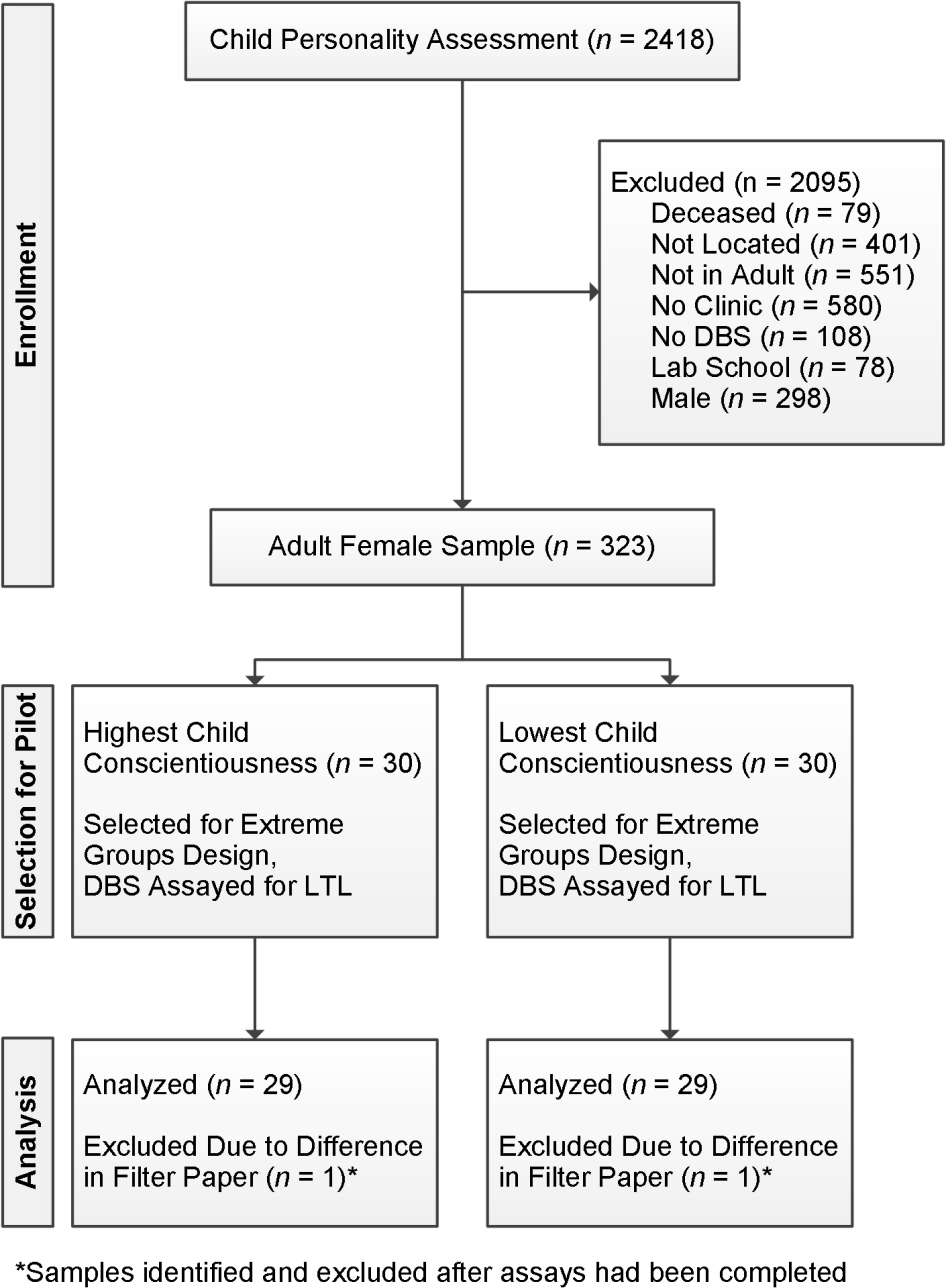
Eligibility and selection of the pilot sample.

Financial constraints limited the number of samples that could be tested in the pilot. To allow for the possibility of finding an association we performed this initial test of the hypothesis that childhood levels of conscientiousness would predict adult LTL using an extreme groups design. LTL tends to differ across sexes, such that women tend to have longer telomeres at any given age [30]. Participants who were assessed in one school, the University of Hawaii Lab School (*n* = 78), were excluded because they were assessed using a different set of items in childhood. Given the concern that sex difference could confound our findings, we elected to limit the current study to only one sex. The pilot sample was selected from women rather than men, and the choice of women instead of men was arbitrary. Fig 1 gives a flow diagram of the selection process for the pilot sample. The subsample for the current study was selected from those adult women who had provided a dried blood spot during the clinic visit and had also consented to allow this material to be used for future genetic analyses (n = 323). We ranked this subset of participants on childhood conscientiousness scores. Of these we selected the 30 scoring highest, and the 30 scoring lowest on childhood conscientiousness. In terms of standardized scores, this corresponded to cutoff points at 1.23 standard deviations above, and 1.40 standard deviations bellow the mean for childhood conscientiousness.

### Measures

#### Childhood personality

For the sample included in our analyses, a common set of 39 items across the samples was assessed across schools. Attributes were selected for their observability and focus groups of teachers developed definitions for each item. For each childhood item, teachers were presented with the definition and a list of their students’ names. At the end of the school year, teachers ranked every student in their class using a fixed nine-step quasi-normal distribution. Students were in grades 1, 2, 5 or 6. The majority of the sample, including all those in the pilot sample, were assessed on only one occasion in childhood. Example items assessing childhood conscientiousness include *Careful of personal belongings*, *Planful*, and *Persevering*. Although the items were selected prior to the widespread acceptance of the Big Five model of personality, Big Five personality factors have been robustly identified in these data, as is described in detail by Goldberg [31]. Of these, the conscientiousness scale demonstrates acceptable reliability with α = .77 [32]. The stability of personality traits tends to increase with age [33]. For a subset of the child sample assessed on more than one occasion, conscientiousness demonstrated adequate one year stability for childhood traits with a test re-test correlation of *r* = .53 [34]. Over long spans of time, personality traits are moderately stable. The best available estimate for the expected stability of personality traits over a 40 year span derived from a meta-analysis of over 150 longitudinal studies of personality is moderate with an estimated correlation of *r* = .25 [33]. Conscientiousness demonstrates a level of stability in our full sample that is directly in line with this estimate. Across a variety of instruments used to collect self-ratings of conscientiousness in adulthood and the teacher assessments in childhood, we find a 40 year stability coefficient of *r* = .25 [34]. In more recent research, teacher ratings of conscientiousness in childhood converge with parent ratings at levels similar to that observed for multiple observers rating adults they know well [35]. Moreover, childhood conscientiousness in the Hawaii study shows predictive validity with respect to outcomes decades later in adulthood including educational attainment [5], the selection of occupational environments [36], health behaviors [5], and objectively measured physical health [26]. This provides strong evidence for the reliability and validity of the teacher assessments.

#### Adult personality

As part of a mailed questionnaire, participants completed 120 items from the International Personality Item Pool (IPIP; items, scoring information, scale reliability, and convergent validity estimates available at http://ipip.ori.org.) designed to assess the Big Five personality factors [37]. Scores on conscientiousness were based on 24 items selected to replicate the facet structure of the NEO-PI-R [38] conscientiousness scale. Participants rated these on a 1 (*not at all like me*) to 5 (*a lot like me*) scale. Adult conscientiousness scores were the sum of these 24 items. Reliability of the IPIP-NEO conscientiousness scale is excellent (α = .90), and the scale shows high convergence with the corresponding NEO-PI-R scale (*r* = .80). The NEO-PI-R is one of the most widely used measures of the Five Factor model and has established reliability and validity [38]. Similarly the IPIP-NEO has demonstrated concurrent and criterion validity [39, 40]. Recent reports linking IPIP-NEO conscientiousness measured in adulthood to health behaviors [41], objective health outcomes [42], and mortality [43] further support the validity of the measure. Ten participants in the pilot sample failed to complete and return the adult personality assessment.

#### Demographics

Demographic variables including date of birth, sex, self-identified culture, and educational attainment were assessed by questionnaire. Educational attainment was measured on a 9-point scale ranging from 1 *(eighth grade or less)* to 9 (*postgraduate or professional degree)*. Five participants did not respond to the educational attainment item.

Participants self-selected their own cultural identity from 12 options, and were instructed to select the one cultural group that they most identified with in cases where individuals identified with more one group. For the current analyses, cultural groups were collapsed into five groups: Caucasian (n = 10), Asian American (n = 21), Hawaiian/part Hawaiian (n = 9), Filipino (n = 11), and other (n = 4). Three participants in the pilot sample declined to indicate their cultural identity.

We performed a series of chi-square goodness of fit tests in order to evaluate whether the observed proportions for cultural groups in pilot sample, and in the selected extreme groups (both high and low) differed from the sample from which these were drawn (n = 323). The proportion of participants in each cultural group did not differ in the pilot sample (χ^2^ = 7.34, df = 5, p = .197) in the low conscientiousness group (χ^2^ = 5.59, df = 5, p = .349), or in the high conscientiousness group (χ^2^ = 9.25, df = 5, p = .100) when compared to the proportions observed in the sample from which these were drawn (n = 323).

#### Smoking

Lifetime history of smoking behavior was assessed via questionnaire. Pack years, defined as smoking one pack a day for one year, were derived from these. To create a smoking variable incorporating cumulative pack years with current smoking status, participants’ self-reported smoking histories were coded into a five point scale based on the distribution of smoking history in the full sample *(0 = ≤ 100 cigarettes smoked*, *1 = previously smoked < 8 pack- years*, *2 = previously smoked ≥ 8 pack years*, *3 = currently smoke ≤ ½ pack a day*, *4 = currently smoke > ½ pack a day)*. Eight participants provided incomplete smoking data.

#### Physical activity

Three questionnaire items assessed strenuous, moderate, and mild physical activity via questionnaire. Participants were asked to rate their activities over a typical seven day period in the past year, including only activities lasting 15 minutes or more. For each item, a list of example activities was provided. Frequency for each item was rated on a five point scale *(0 = zero times*, *1 = one or two times*, *2 = three or four times*, *3 = five or six times*, *4 = seven or more times)*. Scores on these three items were aggregated by a weighted sum. Four participants failed to complete these items. Twelve additional participants were administered a shortened questionnaire that did not include the physical activity items.

#### BMI

Height and weight were recorded as part of the clinic visit using a stadiometer and a balance beam scale. Scores for BMI were calculated using the standard formula (kg/m^2^).

#### Physiological dysregulation

Global health status was indexed using an aggregate score of 11 clinical indicators assessed during the clinic visit. These are systolic and diastolic blood pressure (measured twice and averaged), HDL cholesterol, total cholesterol/LDL ratio, fasting triglycerides, fasting blood glucose, body mass index (BMI), waist/hip ratio, urine protein (log transformed), and whether or not the participant was using prescription drugs for cholesterol or blood pressure.

Participants arrived at the clinic in a fasting state. Blood from a venous arm draw, as well as urine samples, were collected and immediately sent for lab analysis. Waist circumference was measured at the point midway between the lowest rib and the iliac crest. Measures of hip circumference were made at the point where the gluteal muscle showed the greatest protrusion. Blood pressure was measured after five minutes of rest in a seated position using a sphygmomanometer. Two measures were taken with a one minute interval.

Measures were scored such that higher scores corresponded to worse health. For some biomarkers, scores at either extreme can be unhealthy. We evaluated the number of participants in the full sample falling into the low/unhealthy range for each biomarker, and found that in all cases less than 1.2% met this criterion. Each measure was standardized and converted to a deviation score from the mean. The 11 biomarkers were not highly correlated with each other, and factor analysis did not reveal a single factor solution. Following recommendations for aggregating uncorrelated indicators [44] we constructed an index by summing standardized scores, such that higher scores corresponded to greater physiological dysregulation, and worse health. Participant in the full sample who had more than 3 biomarkers missing were not scored on dysregulation. Of the participants included in the pilot sample, all but one had complete data on the dysregulation measure, and one participant was missing 3 biomarkers. More detail on the derivation of the physiological dysregulation scale can be found in Hampson, et al. [26].

#### Leukocyte telomere length

During the clinic visit, blood from a venous arm draw (125 μL) was blotted onto Whatman FTA Micro-cards (cat# WB120210) specifically designed for long-term preservation of dried blood and nucleic acids for quantitative DNA analysis. Following the manufacturer’s instructions, the dried blood spots were air dried, placed in plastic bags with an appropriate desiccant, and stored in the dark at room temperature. Specimens for the current study were identified, then shipped overnight in a room temperature-controlled box to Dr. Côté’s lab at the University of British Columbia (UBC). Researchers who assayed the samples remained blind to all participant data. Six 2mm punches were removed from each blood spot. DNA was extracted from these punches using QIA Amp kit on a QIA Cube (QIAGEN), and LTL was assayed using qPCR following methods described by Cawthon [45] which have been modified and recently extended for use on dried blood spot samples [46]. Samples were assayed in duplicate, on two 96-well plates, using the undiluted DNA extracted from the dried blood spots. The DNA concentrations ranged from 3 to 7 ng/μL. The standard curve was built from a serial dilution of human genomic DNA pooled from frozen whole blood from 24 healthy donors. Three internal controls (one DNA from a human cell line, one from pooled genomic DNA, and the other from a single volunteer) were included in each run and used for quality control related to the Lightcycler run; these were not used to normalize, and their T/S ratio was not incorporated into the standard curve. All samples were amplified successfully and met our quality control criteria. All runs had a PCR efficiency between 1.88 (94%) and 1.95 (97.5%). The intra-run (n-12) coefficient of variation (CV) for this assay is 5%, and based on the internal control included in each of the 22 runs performed, the inter-run CV was 8.5%. The assay produces an arbitrary count for telomeric DNA (T), and for a specific single copy nuclear gene DNA (S). The ratio of these (T/S) was then used as a measure of average relative LTL. We previously showed that DBS LTL is highly correlated with whole blood LTL [46].

### Statistical Analysis

SPSS version 19 was used for statistical analysis. Group comparisons were evaluated using *t*-tests and ANOVAs. Standardized group differences were estimated using Cohen’s *d*. Tests of normality were conducted using the Shapiro-Wilk statistic. Non-parametric tests were conducted using a Mann-Whitney U test. Effect sizes were estimated by Pearson correlations, biserial correlation, and partial biserial correlations where appropriate, and all significance tests (α = .05) were two tailed. To maximize the sample size, all analyses employed pairwise deletion in the presence of missing data, hence sample size varied across analyses.

## Results

Initial examination of the distribution of the study variables revealed two extreme outliers, each with LTL exceeding 3.5 standard deviations above the mean. The two dried blood spots used for DNA extraction for these participants had been stored on filter paper designed for neonatal blood samples rather than the Whatman micro cards used for the rest of the sample. Because these LTL measurements seemed implausible, and the difference could be attributed to the difference in filter paper, they were excluded from our main analysis. Table 1 provides descriptive information on the full sample from which the subsample was drawn, as well as the high and low childhood conscientiousness groups after removing the two extreme outliers, one from each group. The subsample (n = 58) did not differ from the full sample (n = 323) on any of the variables tested, and LTL was normally distributed (*W* = .96, *p* = .09). Consistent with our previous work with the Hawaii cohort, higher childhood conscientiousness was associated with higher educational attainment [5], lower BMIs, and healthier (lower) levels of overall physiological dysregulation [26]. The high and low childhood conscientiousness groups did not differ with respect to age at the time of the childhood assessment, or at the time of the blood draw in adulthood. The two groups did not differ in levels of adult conscientiousness, smoking, or physical activity levels. Due to the use of an extreme groups design based on childhood conscientiousness, childhood conscientiousness was not normally distributed (*W* = .81, *p* < .00). Because of this, associations with childhood conscientiousness were estimated using biserial correlations. The correlation between childhood conscientiousness and adult conscientiousness was similar in magnitude to that observed in our full sample (*r* = .19, *p* = .20, *n* = 48). The correlation was not significant at the level of *p* = .05, likely due the small sample size.

**Table 1:** Sample characteristics of the subsample in comparison to the female clinic sample and for the high versus low childhood conscientiousness groups.

Characteristic	Full Female	Study	*p*	Child Conscientiousness		*p*
	Sample	Subsample		High	Low	
Number of Individuals	N = 323	N = 58*		N = 29	N = 29	
Cultural Identity:						
European American	50 (15%)	10 (17%)		3 (10%)	7 (24%)	
Asian American	142 (44%)	21 (36%)		14 (48%)	7 (24%)	
Hawaiian American	71 (22%)	9 (16%)		2 (7%)	7 (24%)	
Filipino American	34 (11%)	11 (19%)		7 (24%)	4 (14%)	
Other	14 (4%)	4 (7%)		2 (7%)	2 (7%)	
Missing	12 (4%)	3 (5%)		1 (4%)	2 (7%)	
Age at Child Assessment(years)	10.31 (2.1)	9.91 (2.06)	.19	9.80 (2.20)	10.03 (1.95)	.66
Age at Adult Assessment(years)	51 (2.73)	51 (2.53)	.98	52 (2.50)	50 (2.47)	.09
Physiological Dysregulation^†^	-.19 (.49)	-.13 (.61)	.47	.05 (.69)	-.32 (.46)	.02
Educational Attainment^‡^	6.78 (1.79)	6.81 (1.77)	.92	7.58 (.99)	6.07 (2.04)	.00
BMI	228.42(6.66)	29.63 (9.69)	.36	26.65 (7.72)	32.61(10.4)	.02
Smoking^§^	.91 (1.22)	1.18 (1.52)	.25	.92 (1.47)	1.46 (1.56)	.22
Physical Activity**	36.22 (28.80)	39.79 (32.91)	.47	41.66 (30.47)	38.24 (35.41)	.89
Adult Conscientiousness^††^	24.18 (2.78)	24.35 (2.43)	.70	24.78 (2.25)	23.88 (2.57)	.20
Child Conscientiousness	.48 (.61)	.32 (1.57)	.45	1.85 (.21)	-1.20 (.43)	.00
Telomere Length		6.59 (1.28)		6.97 (1.36)	6.20 (1.09)	.02

*Note*. Values reported are means (SD) or n (%). These are provided for the full sample as well as the high and low childhood conscientiousness groups combined and separately. Five participants were missing highest grade, 3 in the high child conscientiousness group and 2 in the low group. Ten participants were missing scores on adult conscientiousness, 4 in the high child conscientiousness group and 6 in the low group. Sixteen participants were missing complete data on physical activity, 10 in the high child conscientiousness group and 6 in the low group. Eight participants were missing data on smoking, 5 in the high child conscientiousness group and 3 in the low group.

*Subsample analyses exclude the two samples stored on neonatal paper noted in the text

^†^Physiological Dysregulation reported in z scores; higher scores indicate poorer health

^‡^Educational Attainment assessed on a 9-point scale where 1 = 8th grade or less; 9 = postgraduate or professional degree

^§^Smoking was coded into five categories (0–4) based on life-time smoking history where 0 = ≤ 100 cigarettes, 1 = previously smoked < 8 Pack years, 2 = previously smoked ≥ 8 pack years, 3 = current smoker at ≤ ½ pack a day, 4 = current smoker > ½ pack a day

**Physical activity is a weighted sum of three items assessing number of instances of mild, moderate, and strenuous exercise in a typical week over the previous year

^††^Adult conscientiousness measured using sums of 24 items scored on a 5 point scale

**Fig 2:**
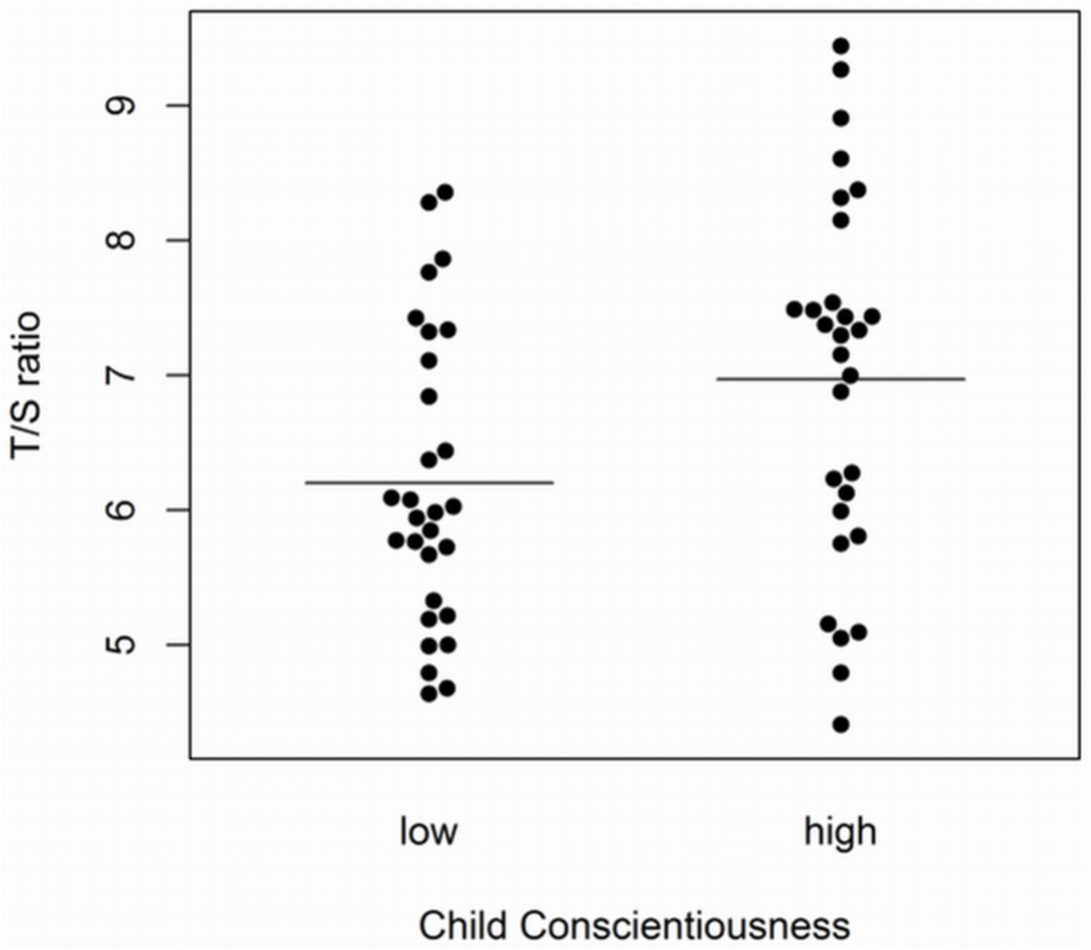
Plots of adult LTL for high and low childhood conscientiousness groups Bee swarm plots illustrating differences in adult LTL (T/S ratio) across extreme groups for high (*n* = 29) and low (*n* = 29) childhood conscientiousness. Group means are displayed as horizontal line.

A *t*-test to evaluate differences in LTL between the two groups was significant (*t* = 2.38, *df* = 56; *p* = .02), such that adult women who had been described by their teachers as higher on conscientiousness 40 years earlier had longer LTL (Cohen’s *d* = .62). This difference is illustrated in Fig 2. We obtained a similar result using a non-parametric test including the two previously removed outliers. Specifically, a Mann-Whitney U test demonstrated that the high childhood conscientiousness group (*n* = 30, median LTL = 7.32) differed from the low childhood conscientiousness group (*n* = 30, median LTL = 6.01) on adult LTL (*U* = 303.00, Z = -2.17, *p* = .03).

We next tested for associations between potential covariates and LTL. Table 2 provides correlations for all study variables and LTL. The only significant correlation reported is the biserial correlation between LTL and childhood conscientiousness (*r* = .30; *p* = .02). Age, physiological dysregulation, educational attainment, and adult conscientiousness were not significantly correlated with LTL.

**Table 2:** Correlations of study variables with LTL.

	*n*	*r*	*p* value
Child Conscientiousness	58	.30*	.021
Age (years)	58	-.16	.222
Physiological Dysregulation	58	-.19	.158
Educational Attainment	53	.21	.132
Adult Conscientiousness	48	.05	.736
Smoking	50	-.03	.850
BMI	58	-.15	.276
Physical Activity	42	.04	.796

*Biserial correlation reported for child conscientiousness and LTL

The subsample did not have sufficient power to use multiple regression to test potential confounding variables or mediators for the effect of interest. None of the potential covariates or mediators in Table 2 were significantly associated with LTL. However, because we expect that these variables may account for some portion of the association between childhood conscientiousness and LTL, we report the partial biserial correlations between childhood conscientiousness and LTL controlling in turn for age, physiological dysregulation, educational attainment, adult conscientiousness, smoking, BMI, and physical activity separately in Table 3. We contrasted these with the raw biserial correlation between childhood conscientiousness and LTL reported in Table 2. Controlling for age did not attenuate the raw biserial correlation, likely due to the narrow age range in the sample. Similarly, controlling for adult conscientiousness had no appreciable effect. Partial biserial correlations controlling for physiological dysregulation and educational attainment tended to attenuate the association. In the case of educational attainment, the partial biserial correlation failed to reach significance (*r* = .27, *p* = .05). Separately controlling for smoking, BMI, and physical activity attenuated the association between childhood conscientiousness and LTL in adulthood in a similar manner.

**Table 3:** Partial biserial correlations between child conscientiousness and LTL controlling separately for age, physiological dysregulation, educational attainment, and adult conscientiousness, smoking, BMI, and physical activity.

Controlling for:	*n*	*r*_*part*_	*p* value	*df*
Age (years)	58	.28	.037	55
Physiological Dysregulation	58	.26	.048	55
Educational Attainment	53	.27	.052	50
Adult Conscientiousness	48	.32	.029	45
Smoking	50	.27	.065	47
BMI	58	.24	.075	55
Physical Activity	46	.31	.052	43

To evaluate the degree to which LTL might vary across cultural groups, we used a one-way ANOVA. We found differences in LTL across groups, *F*(4, 50) = 3.27, *p* = .02. A post hoc analysis using Tukey’s HSD test revealed that individuals who described themselves as Native Hawaiian tended to have shorter LTL in comparison to all other groups (*p* < .05), with the exception of those classified as other. We next tested ethnicity and childhood conscientiousness in a two-way ANOVA using type II sums of squares to partially adjust for unequal groups. The overall ANOVA was significant *F*(9,45) = 3.39, *p* = .00. Both childhood conscientiousness *F*(1, 45) = 5.06, *p* = .03 and cultural identity *F*(4, 45) = 3.04, *p* = .03 remained significantly associated with LTL. There was no significant interaction between ethnicity and childhood conscientiousness.

We additionally tested a series of one-way ANOVAs in order to evaluate the degree to which study variables might have differed across cultural groups. Two variables showed cultural differences. For educational attainment, a one way ANOVA *F*(4, 47) = 3.05, *p* = .026 showed group differences across cultural groups. Post-hoc analyses using Tukey’s HSD showed one significant differences across groups such that Asian Americans (*M* = 7.70, *SD* = 1.35) reported higher educational attainment than participants identifying themselves as Native Hawaiian (*M* = 5.29, *SD* = 1.25). In the case of, BMI a one way ANOVA *F*(4, 47) = 3.05, *p* = .026 showed group differences across cultural groups. Post-hoc analyses using Tukey’s HSD showed that Native Hawaiians (*M* = 35.99, *SD* = 6.35) had significantly higher BMI scores in comparison to Asian Americans (*M* = 25.60, *SD* = 8.43).

## Discussion

To our knowledge, this is the first study to demonstrate an association between a Big Five trait in childhood and LTL in adulthood. Our findings suggest that childhood conscientiousness assessed by teachers predicts LTL 40 years later in adulthood.

Controlling separately for age, physiological dysregulation, educational attainment, smoking, and BMI, attenuated the biserial correlation between of childhood conscientiousness and adult LTL. The association remained significant when controlling for age and dysregulation, but failed to reach significance when controlling for educational attainment, smoking, BMI, or physical activity. While the partial biserial correlation was not significant in this case, the degree to which the observed association was attenuated was small. While it is tempting to view the transition of significant to non-significant as meaningful, this does not constitute a formal test of mediation [47]. Partialing out adult conscientiousness slightly increased the observed biserial correlation (*r* = .32 versus *r* = .30). This difference is likely due to sampling error and the smaller sample size available for this analysis (*n* = 45). Partialing out physical activity similarly increased the magnitude of the observed biserial correlation slightly, although in this case the effect was no longer significant (*r* = .32, p = .05), which is likely attributable to the reduction in sample size due to missing data on physical activity (*n* = 43). We found significant variation in LTL across ethnic groups, such that those identifying as native Hawaiian tended to have the shortest LTL. Both childhood conscientiousness and cultural group identity independently predicted LTL with no evidence of an interaction.

These results suggest a novel pathway linking dispositional traits in childhood to cumulative health that has not previously been incorporated into research on LTL. Childhood personality traits have moderate to low levels of stability across the life-course. Our best estimates of the rank order stability of conscientiousness over 40 years corresponds to a correlation of *r* = .25 [32, 33]. Both trait level conscientiousness and cognitive features of self-regulation develop and improve from childhood to young adulthood [48]. Despite this, childhood conscientiousness predicts outcomes in adulthood including physical health, substance use, career attainment, and financial outcomes [5, 37, 49]. In the broader context of lifespan development, LTL complements the growing list of consequential objective outcomes in adulthood associated with childhood conscientiousness.

We did not find an association between adult levels of conscientiousness and LTL. This must be due in part reality that selecting on child conscientiousness for an extreme groups design magnifies effects for childhood conscientiousness but, given the moderate stability of the trait over 40 years, does not necessarily do so for adult conscientiousness. Sadahiro et al. [25] reported a cross sectional association between conscientiousness and LTL in a sample of young adults at an age intermediate to the two points of personality assessment in our sample. It is possible that conscientiousness assessed at different points in the lifespan may show associations of differing magnitudes with respect to LTL.

Personality and health researchers investigate models of the underlying psychosocial and biological processes that mediate associations between personality traits and health outcomes over the lifespan [50]. LTL assessed in adulthood has several advantages as an objective measure of health status when studying these models: LTL can reflect cumulative insults, changes dynamically over time, and can be readily measured. These features, along with demonstrations that biological stressors, physical morbidity, and subjective states of distress predict LTL, suggest that LTL may be viewed as a psychobiomarker [51]. As such, LTL offers an outcome variable that reflects the accumulation of health effects associated with numerous psychosocial and demographic variables. An important next step for lifespan developmental researchers involves evaluating the degree to which associations with childhood conscientiousness operate independently of adult trait levels and if so, whether these depend entirely on chains of events following from child trait levels and self-regulatory capacities. In models of effects related to health and aging, LTL may play a unique role as an integrative objective outcome.

Particular strengths of the current study are the use of a prospective design with a 40 year interval, and the well-executed assessment of child personality. Although these are preliminary results, they are consequential in suggesting future directions and methods for incorporating LTL research into lifespan developmental models of dispositional traits related to health. Consistent with a lifespan mediation hypothesis, we found that controlling for potential mediators tended to attenuate the strength of association between childhood conscientiousness and LTL.

The current study has specific limitations. An extreme groups design was selected to enhance our ability to detect an association between childhood conscientiousness and LTL in a preliminary sample. However, extreme groups designs do not necessarily produce accurate estimates of effect sizes and tend to inflate effects sizes associated with the selection variable. As a result the raw biserial correlation between childhood conscientiousness and LTL is likely inflated. Similarly, effect sizes for variables correlated with the selection variable can be distorted. In our analyses, partial biserial correlations controlling for variables associated with childhood conscientiousness are likely to be overcorrected. We might expect the true effect size of this effect to be reduced in magnitude in a larger sample. We have secured funding to assay telomere length in our larger sample, and in the future we will test this directly. Additionally, our small sample did not allow us to test multiple competing predictors, account for confounding variables, or test mediation hypotheses. We limited our sample to women, because there is evidence that LTL differs across genders. As a result, our results cannot be generalized to men. Our pilot sample is unlikely to give accurate estimates of effects related to cultural group and these results need to be interpreted with caution. Despite this limitation, our results do suggest the importance of investigating differences in LTL across cultural and ethnic groups, and for controlling for these effects when investigating predictors of LTL. Future analyses using our full sample will allow us to accurately evaluate effects across cultural groups. For all of these reasons, we view the present results as preliminary. Finally, we measured LTL using a qPCR-based assay which may have a higher variability than some other methods but is the appropriate option given the type of sample studied here and the large number of samples to be assayed in the future.

Despite these limitations, the finding of a putative association between childhood conscientiousness and adult LTL is remarkable. There is a paucity of research linking personality at any stage in the life course to biomarkers of health; more so for childhood personality traits. Our results suggest a variety of future directions involving lifespan developmental trajectories of dispositional traits, physical health, and LTL. In addition to providing a more accurate estimate of the effect size of the associations presented here, future research will allow for testing lifespan mediation models where behavioral and environmental mechanisms can be evaluated as explanations of prospective associations between personality traits and LTL. Pathways such as smoking, physical activity, and diet may explain associations between dispositional traits in childhood and LTL years later. Joint or interactive effects of personality traits and sex, socio-economic status, and ethnicity may further explain variability in LTL.
